# Reviewed article: Zago JKM, Anjos GM, McKenna GJ, Leles CR, Jordã LMR. Provision of dental prostheses in the oral health care network: a mixed methods study, Goiânia, Brazil, 2019. Epidemiol Serv Saude. 2026:35;e20250099

**DOI:** 10.1590/S2237-96222026v35e20250099.a

**Published:** 2026-03-27

**Authors:** Elisa Lopes Pinheiro

**Affiliations:** 1 Universidade Federal de Minas Gerais, Belo Horizonte, MG, Brazil

## Peer review A

## Questionnaire

Does the manuscript contain new and significant information to justify publication? Yes

Does the Abstract (Summary) clearly and accurately describe the content of the article? Yes

Is the problem significant and concisely stated? Yes

Are the methods described comprehensively? No

Are the interpretations and conclusions justified by the results? Yes

Is adequate reference made to other work in the field? Yes

## Manuscript Structure

Length of article is: Adequate

Number of tables is: Too few

Number of figures is: Adequate

Please state any conflict(s) of interest that you have in relation to the review of this paper. None

Interest: Good

Quality: Average

Originality: Good

Overall: Good

Recommendation: Major Revision

## Comments

O artigo é muito relevante e contribui para o avanço da ciência e do conhecimento sobre a saúde bucal coletiva. Como ponto alto do artigo destaco o resultado da descrição da rede de atenção representado pela figura esquemática com enfoque no tratamento odontológico em prótese dentária e o envolvimento na pesquisa de três atores sociais fundamentais para a construção do SUS: o trabalhador (dentista), o gestor e o usuário do serviço. Isso, sem dúvida, possibilitou uma maior riqueza de perspectivas sobre a realidade estudada, podendo fornecer subsídios fundamentais para futuras pesquisas mais aprofundadas, para a melhoria do serviço, do processo de trabalho, da gestão do cuidado e da saúde da população. Contudo, este estudo apresenta problemas substanciais que precisam ser cuidadosamente corrigidos. Além disso, o texto apresenta problemas na escrita quanto a clareza, sintaxe, coesão, norma culta. É preciso realizar uma revisão atenta em todo o corpo do texto. Segue minha avaliação detalhada e algumas sugestões para o avanço do manuscrito.

Introdução: É preciso apresentar melhor o problema e estruturar de forma mais consistente a introdução. Por que nasceu a pergunta de pesquisa? Qual a pergunta ou o problema da pesquisa? Após apresentar o objetivo do estudo vocês apresentam o motivo: subsidiar ações de saúde bucal a partir das necessidades em saúde bucal das pessoas. Mas é preciso explicar melhor, extrapolar. Isso já foi feito antes? É inédito? Há uma necessidade percebida (o desconhecimento da provisão e dos fatores que influenciam na provisão de prótese?)? São aspectos muito relevantes para justificar e mostrar o “coração” dessa pesquisa.

1. Melhorar a escrita de modo geral em todo o texto, corrigindo problemas de clareza, coesão, sintaxe, pontuação e regência verbal. Destaco algumas questões para exemplificar:

- nas linhas 45, 46 e 47 da página 2, há uma repetição da palavra “diretrizes” e do termo “saúde bucal”

- nas linhas 8 e 9 da página 3, é possível melhorar a última frase do parágrafo, sugestão: Publicações que dispõem do financiamento dos CEO estão em constante atualização ou estão sendo constantemente atualizadas. Além disso, seria interessante explicar o porquê dessas constantes atualizações: muitas mudanças estão ocorrendo? Por que isso ocorre? Isso influencia na provisão das próteses

- linhas 10, 11, 12, página 3, “(sem vírgula) enfocaram as especialidades mínimas...”. Ex: "Estudos sobre o cumprimento de metas, desempenho e monitoramento da produção dos CEO contemplaram as especialidades mínimas, nas quais a prótese dentária não foi incluída."

2. Sugiro inverter a ordem da introdução, apresentando o problema da perda dentária inicialmente, com suas características, disparidades e singularidades, dados epidemiológicos e a importância de responder/atender às reais necessidades da população. Depois abordar sobre a oferta e a política de saúde bucal, a rede de atenção, como ela se organiza e como a prótese dentária se insere nesse contexto (APS e o CEO).

Métodos: essa seção apresenta problemas e precisa ser cuidadosamente revisada. Recomendo que os autores utilizem referenciais para pesquisas de método misto para melhorar a qualidade e o rigor do relatório de pesquisa. Há uma carência de referencial teórico-metodológico que justifique a escolha do método misto, suporte o delineamento e a condução do estudo, os quais são aspectos fundamentais para o rigor científico. Sugestão de leitura: Cresswell, J. W., & Plano Clark, V. L. (2007). Designing and conducting mixed method research. Thousand Oaks, CA: Sage.; O'Cathain A, Murphy E, Nicholl J. The quality of mixed methods studies in health services research. J Health Serv Res Policy. 2008;13: 92-98. Fundamental seguir uma ordem mais lógica de apresentação metodológica e de fundamentar as escolhas.

1. Delineamento: não está bem explicado, a informação entre parênteses parece mais confundir do que apresentar claramente o delineamento do estudo. A meu ver os autores realizaram uma Pesquisa observacional transversal que utiliza método misto convergente, certo? Acrescentar as referências utilizadas para o delineamento, a justificativa de realizar o método misto. Sugiro reescrever o parágrafo.

2. Contexto:

• Ficou confusa a apresentação do cenário. A oferta de serviços do CEO municipal foi apresentada, mas do CEO estadual não. Por quê?

• A informação do Laboratório de prótese dentária está “solta” entre dois períodos que tratam do CEO.

• As siglas APS, CEO já foram apresentadas na introdução, sugiro já escrever com as siglas...

• É preciso apresentar os distritos sanitários, os quais foram citados na parte qualitativa quando aborda o sorteio.

3. Participante e tamanho do estudo: esse título não representa a totalidade de informações apresentadas nesta seção do método, o qual incluiu além da caracterização dos participantes, da amostra e do local de pesquisa, os procedimentos, a coleta de dados e a análise. Além disso, a metodologia não está clara e rigorosamente documentada.

• Sobre a parte qualitativa: é preciso melhorar a forma de apresentação dos sujeitos, locais de estudo e procedimentos de coleta de dados (forma/critérios de seleção/inclusão, contato com os sujeitos...). sugiro dividir a descrição dos procedimentos de coleta por informante-chave (CD APS, CD CEO, Gestores, usuários).

• Houve critério de inclusão para gestores? Importante informar

• Por que os distritos sanitários campinas-centro e leste foram escolhidos dentre os sete existentes? Explicar os motivos de cada escolha metodológica é fundamental para o rigor da pesquisa.

• É preciso explicar como foi feita e entrevista em grupo com os gestores e o porquê dessa escolha, visto que todas as outras foram individuais. Lembre de referenciar, qual metodologia embasou essa coleta de dados? (Minayo? Moser?) Foi grupo focal ou entrevista em grupo? Justificar a escolha e referenciar.

• É preciso apresentar os roteiros (apêndice/suplementar) e como eles foram elaborados (houve algum referencial?) e por quem foi elaborado (caracterizar melhor a equipe de pesquisadores).

4. Variáveis, fontes de dados, mensuração e métodos estatísticos: é possível apresentar as razões da interrupção dos atendimentos nos dois CEO? Se sim, acrescentar. O parágrafo sobre a integração dos dados é fundamental, mas precisa ser melhor redigido.

Resultados, discussão: Muito interessante! O texto está bem escrito, dando um santo em comparação as outras seções do artigo, e os resultados apresentados são muito importantes para o avanço da ciência e para subsidiar a tomada de decisão. Como sugestão poderia discutir sobre a provisão da prótese total no município, sobre a importância de incluir na carteira de serviços as próteses parciais para a integralidade do cuidado, por exemplo.

A conclusão poderia avançar para além dos achados, refletindo sobre o que este estudo pode contribuir para a melhoria dos serviços, do SUS e para a saúde bucal da população, como já foi iniciado nas recomendações apresentadas nas linhas 38 a 41.

